# Understanding cancer screening participation patterns among first-generation migrants in Germany: a typology based on the intersections of hindering and facilitating factors

**DOI:** 10.1186/s12939-026-02843-w

**Published:** 2026-04-04

**Authors:** Hanna Luetke Lanfer, Doreen Reifegerste, Maryam Efthekhar, Khalida Noor

**Affiliations:** 1 School of Public Health, Bielefeld University, Universitaetsstrasse 25, 33615 Bielefeld, Germany; 2 Independent Authors, Münster, Germany

**Keywords:** Cancer screening, first-generation migrants, Middle East, participation patterns, socioecological model, intersectionality, qualitative study, Germany

## Abstract

**Background:**

Cancer screening (CS) is a core element of preventive care. While international studies highlight that individuals with migration backgrounds often show varied participation in CS, there is a lack of specific evidence regarding first-generation migrants from Middle Eastern countries in Germany. This study aims to bridge that gap by examining CS participation patterns within this group and exploring how individual, social, and structural factors influence these patterns. The focus on Middle Eastern migrants is particularly important due to recent shifts in migration trends, which have led to an increase in the number of individuals from this region relocating to Germany.

**Methods:**

We conducted a qualitative, triangulated study with two datasets: interviews with laypersons (LPs; *n* = 18) who are first-generation migrants, and interviews with healthcare professionals (HCPs; *n* = 17) (first or second generation) across disciplines related to CS; all with ties to Middle East countries. LPs were recruited and interviewed by community researchers; HCPs via physician registries and by academic researchers. Interviews were carried out online or in person, recorded, translated, and transcribed. Analysis followed qualitative content analysis within a socioecological framework. A typology of participation patterns was developed from LP interviews and contextualised with HCP accounts.

**Results:**

CS participation was shaped by interdependent influences across individual, social, and structural levels, including knowledge and perceptions of prevention, language skills and available interpretation services, family roles, network support, organisational procedures, and experiences in care encounters. Comparative analysis of LP and HCP material identified convergences and divergences in how conditions for participation are perceived and navigated. Based on these dynamics, we identified four participation patterns: prevention-oriented, situational, withdrawn/resigned, and marginalised.

**Conclusions:**

Participation among first-generation migrants from Middle East countries is best understood as patterned engagement shaped by intersecting conditions. The typology makes these patterns visible and provides an analytic basis for context-sensitive health promotion. Practically, the study underscores the value of participatory, multilingual approaches in research and the importance of organisational health literacy and communicative and structural competence to support equitable access to preventive services.

**Supplementary Information:**

The online version contains supplementary material available at 10.1186/s12939-026-02843-w.

## Introduction

Cancer screening (CS) constitutes a central pillar of preventive health policy in Germany and plays an essential role in reducing morbidity and mortality through early detection and timely treatment of malignancies [1]. The German healthcare system offers a range of population-based screening programs, including those for breast, cervical, colorectal, and skin cancer, all of which are structured by statutory regulations and stratified according to age, biological sex, and epidemiological risk. Participation in these programs, however, is not evenly distributed across the population. Growing evidence indicates that individuals with a migration history participate less frequently in cancer screening compared with the autochthonous population [2, 3]. This disparity has far-reaching implications, as lower participation rates are associated with later-stage diagnoses and poorer prognostic outcomes [4].

Similar participation differences have been reported in various other national contexts. Existing international studies offer valuable, albeit inconsistent, insights into how migration-related factors influence screening behaviour. They repeatedly demonstrate that participation is contingent upon a multidimensional interaction between language proficiency, health literacy, cultural and religious beliefs, social norms, family arrangements, and previous encounters with healthcare professionals and institutions [5–9]. Moreover, a meta-analysis with 41 included studies from different high-income countries documents inequities across the continuum of cancer-related healthcare, even within groups with similar cultural backgrounds [4]. International evidence also suggests that structural factors of healthcare systems (e.g., how access is organized, information is distributed, and preventive care is embedded) hinder and facilitate the conditions of participation [10, 11]; hence, understanding these dynamics in Germany requires closer examination within the German healthcare system. To situate our study, we therefore briefly outline three context-specific issues that frame the conditions of CS participation in Germany.

First, migrants who arrive from healthcare systems characterized by different, less formalized access mechanisms may find institutional arrangements confusing or exclusionary as screening offers in Germany are stratified by age and gender according to epidemiological risk assessments. This stratification, while epidemiologically justified, creates differentiated entry points that presuppose familiarity with bureaucratic processes and preventive care logics characteristic of the German system [2]. Moreover, the system’s reliance on precise procedural steps, including the need for referrals and understanding of statutory versus private coverage, poses additional challenges for migrants unfamiliar with these intricacies. International findings can only be transferred to a limited extent, as each healthcare system operates within its own institutional logics and access structures.

Second, at the moment, the Federal Statistical Office [12] uses migration history as a statistical umbrella term that encompasses both first- and second-generation migrants whose parents immigrated from a wide variety of countries and which refers to approximately 25.6% of the German population. This wide category fails to capture important distinctions in migration trajectory, legal status, language proficiency, and healthcare socialization [13]. The diversity of experiences, which blurs expectations, and barriers that shape interactions with preventive healthcare services of different subpopulations, highlighting the need for more granular, differentiated analyses within and across migrant groups.

Third, while there is increasing research with and on different subpopulations and their CS participation [14–16], these studies have focused (a) primarily on migrants from Türkiye and the former Soviet Union with long-standing migration histories in Germany and (b) on women and gender-specific screening offers, i.e., breast and gynaecological screening. These studies have provided valuable insights into participation dynamics within these groups but offer only limited understanding of broader mechanisms shaping engagement with CS across different populations. Owing to geopolitical upheavals and armed conflicts, the number of individuals migrating from Middle Eastern countries to Germany has increased substantially in recent years [13], yet empirical research on their participation in CS within Germany remains extremely limited.

Building on these gaps, our study seeks to broaden the analytical perspective by examining the interconnections between individual, social, and structural factors that influence CS (non-)participation of first-generation migrants. To this end, our study turns to the socioecological model as a conceptual framework. Rooted in Bronfenbrenner’s ecological systems theory [17] and later adapted for public health and health promotion [18, 19], the model conceptualizes health behaviours as emerging from the interplay of multiple, interdependent levels of influence. At the individual level, it directs attention to factors such as health beliefs, prior experiences, and embodied knowledge that shape perceptions of cancer risk and preventive care. At the social level, it may show gender norms, community discourses and other social factors that can either silence or support engagement with CS. At the structural level, it foregrounds the organizational designs, access logics, and professional practices of the healthcare system itself, asking how institutional infrastructures enable or inhibit participation. In deploying this multi-level lens, our study seeks to understand the interlocking factors to participation in CS:

RQ1: Which individual, social, and structural factors influence (non-)participation in CS among first-generation migrants from Middle East countries in Germany?

While studies of CS (non-)participation have provided important insights, they tend to conceptualise participation in binary terms; that is, whether someone takes part in screening or not. Yet recent research points to complex patterns of participation which may change over time and are shaped by differences in social positioning such as gender and caregiving responsibilities [20, 21].

Hence, beyond identifying individual, social, and structural factors, the study seeks to understand patterns in participation and therefore develops a typology. Typological approaches have a long tradition in sociology and qualitative health research [22, 23] and they provide a systematic way to synthesize complex, multi-level data into patterns. In the context of CS, a typology can reveal how a combination of factors – ranging from sociodemographic factors such as gender to health beliefs, social roles, and structural access conditions – interact to shape patterns of CS (non-)participation [24]. This approach aids in understanding the spectrum of migrant experiences with CS. It may also inform targeted interventions that accommodate the diverse needs and orientations of different migrant subgroups towards CS, thereby enhancing the effectiveness of health promotion strategies.

RQ 2: Which types of CS (non-)participation can be distinguished among first-generation migrants from Middle East countries in Germany?

## Methods

### Study design

This study is part of larger, participatory project on culturally sensitive health information on CS for first-generation migrants from the Middle Eastern region [25, 26]. It employed a qualitative, triangulated design, drawing on two distinct groups of migrants to capture a broad spectrum of experiences with CS in Germany: (1) first-generation migrants without a professional healthcare background (hereafter abbreviated to LP for layperson) and (2) migrants, including both first- and second generations, with a medical degree in a medical discipline related to CS (hereafter abbreviated to HCP for healthcare professionals). This design allowed us to explore the perspectives of both laypersons and healthcare professionals, while considering how migration histories, professional experiences, and social contexts shape engagement with CS.

### Sample and recruitment

For the first group of laypersons, a total of 18 LPs were interviewed. Participants were recruited in close collaboration with four trained community researchers who are themselves first-generation migrants from a Middle Eastern country (see Table 1 for an overview of community researchers and their recruitment networks). The community researchers did not know each other before this study and lived in three different places in Germany. The community researchers received between 250 and 1,000 Euros for their work, depending on the length and engagement with the project. While two community researchers participated throughout the whole study (development of the interview guide, pretesting, translation of the interview guide, recruitment, data collection, data translation, and analysis), two were involved in certain steps (commenting on the interview guide, translating it, and contributing to recruitment, data collection, and translation).

**Table 1:** Overview of community researchers and recruitment networks

Gender	Country of origin	Number of interviews conducted	Countries of origin represented among interviewees	Notes on recruitment networks
Female	Iran	6	Iran	Personal and religious networks
Female	Afghanistan	4	Afghanistan	Personal networks
Male	Türkiye	3	Türkiye	Family and personal networks
Female	Türkiye	5	Türkiye, Iran	Personal and online networks

Inclusion criteria specified that participants had migrated to Germany from a country in the Middle Eastern region within the past ten years (see Table 2 for demographics). Interviews with LPs were carried out in two waves between December 2024 to January 2025 and May to June 2025 and conducted either in person or online, in the participants’ native language or a language in which they felt comfortable. Interviews were conducted by one of the community researchers, occasionally with the first author present. Interviews lasted between 30 and 85 min, and each participant received 30 Euros as compensation. Written informed consent was obtained from all participants; for some, consent was documented under a pseudonym to address their concerns about potential implications for their legal or asylum status. Interviews covered participants’ health behaviours and preventive practices, experiences with the German healthcare system, trust and communication with healthcare professionals, information and support needs, and the influence of migration and family responsibilities on access to cancer screening (see the interview guide in the supplementary file).

**Table 2:** Sociodemographic characteristics of layperson migrants *(N* = 18)

Characteristics		*n*	%
Gender			
	Female (F)	9	50
	Male (M)	8	44
Age (grouped)			
	18–29 years	4	22
	30–39 years	7	39
Country of birth			
	Afghanistan	4	22
	Iran	7	39
	Türkiye	7	39

For the second group of health professionals, 17 HCPs were recruited by HLL and two student assistants (see Table 3 for demographics). Recruitment was facilitated by querying databases of the Association of Statutory Health Insurance Physicians (in German: Kassenärztliche Vereinigungen) in several German federal states to identify physicians with relevant language skills from Middle Eastern countries and disciplines associated with cancer screening, such as gynaecology, urology, dermatology, gastroenterology, and general internal medicine. Identified physicians were contacted sequentially by email to ensure diversity across medical disciplines and language backgrounds (see Luetke Lanfer et al., [25] for a description of the recruitment approach). Interviews were conducted either online or in person between November 2023 and January 2024 by HLL and a student assistant. They lasted between 37 and 72 min, and participants received 70 Euros for their time. This higher reimbursement reflects the professional context of these participants. All HCP participants provided written informed consent prior to the interviews. Interviews addressed professional experiences with cancer screening among migrant patients, perceived barriers and facilitators of participation, communication and trust, access to and quality of health information, and professional reflections on improving preventive care structures (see supplementary file).

**Table 3:** Professional and sociodemographic data of HCPs (*N* = 17)

Characteristics		*n*		%
Gender				
	Female	9		53
	Male	8		47
Professional discipline				
	General practice/Internal medicine	6		36
	Gynaecology	5		28
	Dermatology	2		12
	Urology	2		12
	Pneumology	1		6
	Gastroenterology	1		6
Migration background (first and second generation)				
	Syria	5		28
	Iran	4		24
	Türkiye	3		18
	Palestine	2		12
	Iraq	1		6
	Afghanistan	1		6
	Jordania	1		6
Clinical setting/place of work				
	Outpatient/ambulant	10		59
	Inpatient/hospital	7		41

### Data analysis

All interviews were audio-recorded, translated, and transcribed using AI services (f4X), with human checks for accuracy. Data were analysed using qualitative content analysis [27], supported by MAXQDA (VERBI Software, 2022). Due to our triangulated study design, the two datasets (LPs and HCPs) were analysed separately in the first step. For our first research question, the analysis combined deductive and inductive coding: deductive main categories were derived from the socio-ecological model, structuring the data around individual, social, and structural levels of influence on CS participation, while inductive subcategories were iteratively developed through close reading of the transcripts to capture additional themes emerging from participants’ narratives. The three levels served as heuristic categories to structure the data, though boundaries between them were not always clear-cut. For example, material was coded as individual when participants described a personally perceived experience (e.g., feelings of shame, trust, or fear). By contrast, data were coded as social or structural when participants referred to broader dynamics (e.g., observed discrimination).

To ensure rigor and consistency, a consensus coding approach was applied. The first author conducted initial inductive coding on a subsample of interviews to generate preliminary categories, which two student assistants then applied to the same material. The first author reviewed their coding, consolidated overlapping or redundant categories, and documented discrepancies. These were discussed, resulting in a refined coding frame. Using this finalized frame, the student assistants coded the remaining interviews, and the first author reviewed and re-coded each transcript to ensure consistency and analytic coherence. In a second step, findings from the two datasets were systematically compared to identify converging and diverging themes, as well as complementary insights across the groups.

For the second research question, we aimed to identify empirically grounded typologies of participation and non-participation in CS. Building on the results of RQ1, we focused on the dataset of LPs and used their interviews as the basis for typology development. In a first step, all LPs were grouped according to their reported participation patterns, distinguishing between (a) regular, (b) irregular, and (c) no participation in any cancer screening program. This initial grouping served as a structural starting point to explore how different degrees of engagement related to individual, social, and structural experiences described in the interviews. In a second step, these groups were further refined through within- and cross-case comparisons that integrated relevant sociodemographic characteristics (e.g., gender, age, occupation in Germany) and migration histories. Because we identified two distinct groups among irregular participating interviewees, we developed two additional sub-types, resulting in a typology consisting of four groups. In the final step, interviews from HCPs were revisited to validate and contextualize the typology, i.e., how healthcare professionals’ perspectives aligned with or contextualized the orientations observed among LPs.

AI (ChatGPT 5 by OpenAI) was used for language and grammar refinement upon completion of the manuscript.

## Research team and reflexivity

The research team consisted entirely of women with different professional and biographical backgrounds. HLL led the study, coordinated the overall design, and conducted most interviews with participants who had a professional healthcare background. Together with DR, she developed the study design and led data analysis, interpretation and manuscript preparation. Both HLL and DR work in academic research and have no personal migration experience. Their positionality as university-based researchers informed the design and analytical framing of the study, while they remained aware of their outsider perspectives regarding the lived experiences of migrants in Germany. ME and KN, both first-generation migrants with academic degrees and professional experiences outside academia, worked as community researchers. They were closely involved throughout the entire research process. Their linguistic and cultural proximity to participants helped to establish trust and provided essential contextual understanding for this study. Regular team debriefings throughout data collection and analysis allowed us to critically reflect on how our respective positionalities shaped the research process and to ensure that interpretations remained grounded in participants’ perspectives.

## Results

### Individual, social, and structural factors influencing (non-)participation in CS (RQ1)

To address RQ1, we used a socioecological model with three levels – individual, social, and structural – each comprising facilitating and hindering factors. This section presents a condensed synthesis highlighting the most salient patterns and interpretive links across levels whereas detailed categories, illustrative quotes, and the full coding structure are provided in Table 4.

**Table 4:** Illustrative quotes for facilitating and hindering factors across individual, social, and structural levels

Level	Subcodes	Sample quote
Individual level		
Facilitating factors	Trust in the German (healthcare) system	“With every illness, I have the feeling that Germany not only supports me in treating the illness and during the illness process, but also in the rest of my life. The welfare state, the healthcare system, the insurance system, gives me an enormous sense of security.” (LP, Türkiye, M40)
	Perceived respectful treatment in healthcare encounters	“But one thing I really like about Germans: no matter the situation they are in, even if they feel bad, they say ‘Good morning’ or ‘Good day’ when they see you. They come with a smile to the door of the waiting room to pick up their patients. Yes, I like that, and many of them are truly friendly and kind-hearted people by nature.” (LP, Iran, W50)
	Knowledge and awareness about preventive services	“I’m naturally someone who cares a lot about my health. For example, two years ago I went for a mammography. There they only did a manual exam and said everything was fine. But I insisted that they also do an ultrasound. They said that at my age it’s not necessary, it’s only recommended from age 50. But I said I heard it can also be done for younger women and I wanted to be sure that everything was fine. What I want to say is: when I hear about something or when it seems important, I follow up.” (LP, Iran, W48)
	Socialization with preventive healthcare in country of origin	“Let’s take the example of skin cancer screening: from my experiences in Türkiye, I had a rough idea of what to expect because I went there, not every year, but maybe two times. But some doctors here [Germany] did the examination much more thoroughly than I had expected. That surprised me.” (LP, Türkiye, M32)
	Strong preventive health behavior in everyday life	“For my physical health, sports have always been part of my life. I’ve been doing sports since I was 12 years old. I train every day, not too long and not professionally. My goal is not to achieve a specific figure, lose weight, or have a six-pack. My goal is to stay healthy, physically and mentally.” (LP, Iran, W48)
	Religious or cultural beliefs that promote prevention/health consciousness	“Germans should know how important religion is for us, especially during Ramadan. I don’t think it should always be seen so negatively. There is care in this, taking care of the body and it should be acknowledged.” (HCP Gynaecology, Syria, W)
Hindering factors	Lack of trust in the German (healthcare) system	“Excuse me for saying this, but I have the feeling that German doctors are a bit anxious and cautious. They seem afraid to make a diagnosis right away. They rely a lot on machines and technologies. But in Iran, with my experiences there, I have the feeling that the doctors are braver, stronger, and more decisive when making a diagnosis and recommending treatment.” (LP, Iran, W41) “After all the experiences I’ve had, no, I don’t trust them. First, as I said, they are very slow. They don’t take things seriously at all. A month ago, I was sick, but out of fear that the doctor would just say, ‘Drink tea,’ I didn’t go. It has already happened to me two or three times that I was seriously ill and the doctors didn’t help me. They just sent me home, and I came back in a worse condition. Doctors think they are saving costs for the insurance, but in reality, they are increasing costs. Patients get sicker and put more burden on society. That’s a problem in the medical system.” (LP, Iran, W45)
	Perceived dismissive/discriminatory treatment in healthcare encounters	“He wasn’t very friendly and just told me to drink water and that it’s just how it is, we don’t give medication. He said, ‘We’re not like you, who always take medication.’ That was my experience with some doctors here.” (LP, Iran, W41)
	No/limited knowledge and awareness about preventive services	“A German woman knows she can go for early detection exams every year, not because she read it somewhere, but because she sees it around her – friends, family, others. Migrants don’t have those kinds of contacts; most of their networks are with other migrants, so nobody knows much about it.” (HCP General Practice, Türkiye, M)
	No socialization with preventive healthcare in country of origin	“Arab mentality is different. People go to the doctor only when they have complaints.” (HCP Gastroenterology, Jordania, M)
	Competing life priorities	“Many have depression, psychological problems, homesickness. Nobody flees voluntarily. These are often tragic fates. Women who haven’t seen their mothers for six, seven years, or left their children behind. That’s a heavy burden. Of course, everything becomes less important if you have such worries on your mind and thinking about a non-existent illness is off your mind.” (HCP Gynaecology, Syria, W) “Why I don’t do preventive care – that’s a good question. I don’t have a clear answer. Maybe we can just call it life. Because of the intensity of my private life, at work, I try to keep everything in balance.” (LP, Türkiye, M56)
	Feelings of shame and vulnerability during screenings (e.g., gynecological screening)	“What also bothers me: when you have to undress for an exam at the gynaecologist, for example, you don’t get a blanket to cover yourself. That makes me feel very vulnerable and uncomfortable.” (LP, Türkiye, W36)
	Fear of illness and avoidance of knowing about it	“Many simply don’t want to find out if something is wrong. They think it would only make life harder. So they prefer not to know.” (HCP General Practice, Türkiye, W)
	Poor German language skills	“I never took language classes in Germany though I wanted to. My German, you can see, I can’t say much … The doctor, gynaecologist, has to explain everything and says I have to come with a translator. But I don’t have anyone to come with me, so I don’t go.” (LP, Afghanistan, W32)
**Social level**		
Facilitating factors	Perceived safety and social stability in Germany	“I feel safer in Germany … I can care about things like my health. That was different in Türkiye before.” (LP, Türkiye, M32)
	Fewer taboos, especially on sexual topics.	“I can openly talk about love and that stuff (laughs). This makes a big difference if you can be more open about things!” (LP, Türkiye, M34)
	Changing gender roles (greater freedom for women)	“Overall, I think Germany has given women more opportunities to become independent. Even if I had stayed in Iran, I wouldn’t have had so much independence. But now I have more independence. I have more peace and can make my own decisions.” (LP, Iran, W50)
	New and existing social networks (physical and digital)	“Iranians in Germany are very well connected, there are Facebook groups and WhatsApp groups. So news gets around quickly. People also spread information like, there is a Persian speaking doctor in this city and then everyone within 50 km or more goes there.” (HCP Pneumology, Iran, W)
	Language mediation as a cultural bridge	“In their first years, most people will have to rely on language mediation when they see doctors. For me, this can be a chance because a good language mediator becomes aware of the things that are not necessarily said but can still lead to misconceptions. In that way, a language mediator can help a patient also understand about the culture and vice versa.” (HCP Gynaecology, Palestine, M)
Hindering factors	Gender hierarchies and socially shaped gender roles	“When I came here, the husband didn’t want me to go outside. Not to talk to anyone or make friends, always to stay at home like in a prison. Not to go out. He said if you go out and meet someone else, especially Germans, you’ll understand your rights and you will get worse. You will forget your Afghan life. You will forget your Islam and you lose yourself. Yes, and that’s why I stayed at home. He also forbade me to go to language classes. He said you’ll become a bad woman. He said bad things, really bad words.” (LP, Afghanistan, W32)
	Isolated social networks	“They are connected, but only with their own people, not with Germans. That, of course, is also a barrier for speaking and for information. Because they speak Arabic with each other, or they talk to people from Hamburg, Berlin, cousins from here and there, but always within their own people.” (HCP Gynaecology, Palestine, M)
	Lack of support from close family members	“Families are also bigger where we come from … And that can influence the decision about whether a patient does an exam or not by 70–80%. The whole family gives their opinion.” (HCP General Practice, Syria, M) „For women, it’s especially hard that their own family, sister, mother, and so on are not here. Because they often have few contacts and are alone.” (LP, Afghanistan, W52)
**Structural level**		
Facilitating factors	Organizational features of the healthcare system (e.g., technologies, good training of physicians, professional liability and clinical guidelines)	“The equipment is modern and the medical staff is usually very attentive – although, of course, there are differences, like everywhere.” (Türkiye M28) “I really, really like that they talk honestly with you. I find it really great that no matter what happens, you must understand it and they explain everything until you do.” (LP,Iran, M38)
	Financing mechanisms (coverage of costs for screenings by statutory health insurance)	“Here, when you go to the doctor, you don’t have to worry about insurance and you don’t have to worry about whether you have enough money in your pocket or how expensive the medication will be. You have a certain security. You feel financially calm because the insurance covers many of these things. … You can actually care about prevention because it’s also covered.” (LP, Iran, W48)
	Cultural sensitivity & language diversity (due to increasing number of healthcare professionals with a migration background)	“Not all doctors in Germany are Germans. In Berlin, they are actually the minority. That’s an advantage for me.” (LP, Türkiye, M32) “And when I sometimes see that patients really struggle and try to communicate with hands and feet, trying in German because they don’t know I speak Arabic, then I’m glad I can just switch to their language. It’s such a terrible feeling to know, I’m in pain or something is wrong, and I can’t express myself.” (HCP Dermatology, Türkiye, W)
Hindering factors	Systemic unequal treatment in healthcare	“Another prejudice from German doctors is that they think patients with a migration background, especially from the Mediterranean or the Middle East, tend to exaggerate their suffering. Like, they think these patients play the victim, show more pain, and demand help right away. Whereas if a European patient had the same illness, they would behave more ‘normally.’ This has even been proven scientifically. But I think it comes from cultural, social, or psychological stress factors — that these patients live in a completely different culture and society and have to cope with that. I think that also has a negative impact, making them more sensitive when it comes to pain or serious illnesses.” (HCP Gynaecology, Palestine, M) “Another thing is that doctors sometimes make a lot of assumptions when they give information, and they draw conclusions based on stereotypes. I’ve honestly experienced that too.” (LP, Türkiye, M34)
	Organizational features of the healthcare system (e.g., bureaucratic barriers, complexity of structures and long waiting times)	“Because of the language barrier and the complexity of the systems, I always worry that I will react too late in some health situations. That maybe I could have prevented something or gotten a treatment in time, because of lack of information, lack of confidence, or frustration with the system, I’m too late and at a disadvantage.” (LP, Türkiye, W34) “Getting an appointment with specialists is almost impossible. For example, I urgently need a cardiology appointment, but I haven’t received a response to my request for months. You can’t get through on the phone either.” (LP, Türkiye, M56)
	Limited institutional support for multilingual communication	“How is she supposed to understand anything without language skills? For most things, you just can’t explain that without language mediation. In many hospitals, there are doctors and staff with different languages, but in practices, it’s harder to implement and there is no legal right to language mediation. Not everyone has close people around them whom they can or want to take along for translation. And honestly, I don’t have a solution; you can’t offer every language in every practice, it’s impossible.” (HCP General Practice, Türkiye, W)
	Financing mechanisms (uncertainty about out-of-pocket vs. covered services by statutory health insurance)	“Migrants often don’t know how health insurance works. I often hear from people: ‘Okay, I went to the doctor, he only wanted money from me and took a lot of money,’ even though the insurance pays, and they think the doctor did it on purpose. They need to understand that not everything is free. I see very, very little information available for this group.” (HCP General Practice, Syria, M)
	Cultural insensitivity of healthcare providers	“I remember a scene. A German doctor comes in, stays completely neutral, and says, ‘You have breast cancer at this stage. These are your options: this, that, and the other.’ And that’s it. A Turkish doctor, on the other hand, comes in with emotion and body language: ‘Let’s talk for a moment.’ Of course, the German doctor might say the same things, but we expect more reaction and dialogue. I really try, when giving a diagnosis, to convey more empathy. I speak slower, I give more opportunities for the patients to express themselves.” (HCP General Practice, Türkiye, W)

#### Individual level

*Facilitating factors.* At the individual level, knowledge and awareness about preventive services and system structures emerged as enablers of participation. Both LPs and HCPs described that familiarity with the organization of preventive services increased confidence and access. Strong preventive health behaviour in everyday life was another facilitating factor, characterized by proactive health-seeking and regular engagement with preventive offers. Religious or cultural beliefs that promote prevention were also noted, particularly when preventive care was perceived as part of moral responsibility or self-care.

LPs emphasized that prior socialization with preventive healthcare in the country of origin lowered emotional barriers and made screening appear familiar, while HCPs highlighted trust in the German healthcare system as a product of consistent and respectful encounters, which reinforced preventive behaviour.

*Hindering factors* at this level included limited awareness or uncertainty about available screening programs and no prior socialization with preventive healthcare in the country of origin, leading to uncertainty about the rationale for screening. HCPs frequently linked non-participation to unfamiliarity with preventive logics and culturally shaped expectations to seek care only when symptomatic. LPs described feelings of shame and vulnerability during intimate examinations, fear of illness and knowledge about it, and competing life priorities such as work, migration-related stress, and administrative burdens. Both LPs and HCPs referred to religious or cultural beliefs that discourage prevention, including fatalistic views of health and illness. Experiences of discrimination or perceived dismissive treatment were described almost exclusively by LPs and were identified as critical barriers undermining trust and continuity of care.

#### Social level

*Facilitating factors.* Perceived safety and social stability in Germany were a recurring enabler, hence, creating a sense of security necessary to engage in long-term health behaviour. Both LPs and HCPs associated fewer taboos, especially on sexual topics, with greater openness toward preventive care. Changing gender roles were described as facilitating factors by LPs, particularly when migration increased women’s autonomy in healthcare decision-making. HCPs confirmed this trend but noted that it varied across family contexts. New and existing social networks (physical and digital) provided crucial informational and emotional support, while language mediation through informal contacts functioned as a pragmatic bridge to access preventive services in the absence of professional interpreters.

*Hindering factors* at this level included isolated networks, where strong intra-community ties limited exposure to preventive health information. HCPs described gender hierarchies and socially shaped gender roles as continuing barriers, restricting women’s ability to access gynaecological services independently. LPs additionally emphasized a lack of support from close family members, particularly in households with collective decision-making norms. HCPs noted that these relational dynamics could significantly influence whether individuals acted on screening recommendations.

#### Structural level

*Facilitating factors.* Both datasets identified structural conditions that enabled participation, including good technologies in healthcare provision and high professional training standards in Germany, which enhanced trust in medical institutions. Coverage of costs by statutory health insurance was described as a decisive factor removing financial barriers common in other systems. The increasing number of healthcare professionals with a migration background was also seen as facilitating communication and cultural understanding. LPs frequently highlighted friendly and respectful treatment as an institutional feature that encouraged participation, while HCPs referred to good explanations for examinations as a structural expectation embedded in German healthcare.

*Hindering factors.* Several systemic barriers were identified. Both LPs and HCPs mentioned limited institutional support for multilingual communication, particularly in outpatient settings, as a major obstacle to access. Barriers due to bureaucracy and the complexity of healthcare structures were described as deterring engagement, especially for newcomers unfamiliar with administrative processes. Uncertainty about out-of-pocket vs. covered services created suspicion toward medical providers. HCPs further emphasized a high workload and time constraints of providers as institutional challenges that limited patient-centred communication. Finally, both LPs and HCPs pointed to lack of cultural competence in medical training and systemic unequal treatment in healthcare as structural barriers reducing trust and willingness to participate in preventive screening.

### Types of CS (non-)participation (RQ2)

The following section presents four types of CS (non-)participation derived from the LP dataset. Each type summarizes shared characteristics and recurring patterns across participants. Table 5 provides an overview of detailed participant sociodemographic data and how they were distributed across the four types.

**Table 5:** Distribution of LP participants across participation types

Gender	Age	Country of origin	Length of stay in Germany	Work status	Marital status, living situation	Language skills*	Health condition	
Prevention oriented (regular participation)								
Female	50	Iran	Since 2015	Paid work	Married, 1 adult child	Very good	No own health burdens, but partner has	
Male	32	Türkiye	Since 2017	Paid work	Single, living alone	Very good	Past and current health burdens	
Male	27	Iran	Since 2021	Paid work	Single, living alone	Good	No health burdens	
**Situational participation (irregular participation**								
Male	22	Türkiye	Since 2017	Paid work (part-time)	Single, sharing flat	Very good	No health burdens	
Male	28	Türkiye	Since 2017	Paid work	Single, living with parents	Very good	No health burdens	
Male	56	Türkiye	Since 2017	Paid work	Married, 2 adult children	Good	Current health burdens	
Female	48	Iran	Since 2019	Paid work	Married, 2 minor children	Good	No own health burdens, but child has	
Male	38	Iran	Since 2020	Paid work	Married	Good	No health burdens	
Female	36	Türkiye	Since 2018	Paid work	Married	Fair	Current health burdens	
Male	34	Türkiye	Since 2017	Paid work	Single, living alone	Very good	Current health burdens	
**Withdrawn or resigned (irregular participation)**								
Male	39	Iran	Since 2019	Paid work	Single, living alone	Good	Past health burdens	
Female	45	Iran	Since 2018	Paid work	Single, living alone	Very good	Past and current health burdens	
Female	41	Iran	Since 2019	Paid work (part-time)	Married, 1 minor child	Good	Past and current health burdens	
Male	40	Türkiye	Since 2017	Unemployed (temporary)	Single, living alone	Very good	Past and current health burdens	
**Marginalised (no participation)**								
Female	32	Afghanistan	Since 2015	Unpaid care work	Divorced, 4 minor children	Basic	No health burdens	
Female	28	Afghanistan	Since 2022	Unpaid care work	Widowed, 2 minor children	Basic	No health burdens	
Female	35	Afghanistan	Since 2018	Unpaid care work	Married, 4 minor children	Basic	Past health burdens	
Female	52	Afghanistan	Since 2019	Paid work (part-time)	Married, 2 adult children	Basic	Current health burdens	

*Self-reported language proficiency at the time of the interview, classified according to the Common European Framework of Reference for Languages (CEFR): “basic” (A1–A2), “fair” (B1), “good” (B2), and “very good” (C1–C2)

#### Type 1: Prevention-oriented (regular participation)

Prevention-oriented participants in our sample were usually mid-aged (30–50 years), both men and women, and with employment in Germany. They lived either with partners or alone and had sufficient German language skills (at the time of the interview) to navigate the healthcare system independently. For them, CS was part of a broader, health-conscious lifestyle:

There are so many apps you can use for your health. When I go for a walk, my phone counts every step – that gives me such a good feeling. The apps also helped me realize which foods aren’t good for me and why I was gaining weight. Now I’m more mindful about what I eat. I also do meditation for my mental health, so I feel I can do a lot for my wellbeing myself. (LP, Iran, W50)

Participants assigned to this group were well-informed about available service and some actively asked for them. Several had already been socialised with preventive healthcare in their country of origin, which helped them to understand how such examinations worked. Others described that past health problems, either their own or in their families, had sharpened their awareness of prevention. Their encounters with the German healthcare system were ambivalent to positive: they trusted their physicians (or at least one) and valued the structured training system of German medical professionals, which reassured them of medical competence. Nonetheless, they also voiced frustration about bureaucracy or long waiting times which required long-term planning to participate in CS:

The whole process with appointments is really annoying. You basically have to know six months in advance when you want to see a doctor. (LP, Iran, M27)

#### Type 2: Situational (irregular participation)

Situational participants were the largest group in our sample. They were often younger (in their twenties or thirties), more frequently men, and usually either employed or studying. They lived with partners, parents, in shared flats, and their German language skills were generally good to very good. Their participation in CS was irregular and dependent on life circumstances. They might attend an examination when a doctor asked them to, when a family member or close friend was seriously ill, or when a specific symptom made them worry. Most described themselves as healthy and therefore saw little urgency for screening. Moreover, they were busy balancing work, studies, family demands and building a life for themselves after migration, hence prioritizing other demands in life:

I didn’t come here by choice but had to leave for compelling reasons. Of course, that had a strong impact on my mental health. I had a good life in Turkey and had to start from zero here. That wasn’t easy. I had to learn the language, find work – that was my main focus in the first years. I knew I had to get my life back under control as quickly as possible. (LP, Türkiye, M56)

Some had also experienced that there were few offers for them to participate in any screening as these are usually tied to older age. Their knowledge of preventive services was basic, but they knew how and where to access information about CS. Their encounters with the health system were mixed: some valued their family doctor, while others described confusion about referrals and uncertainty about what was covered by insurance. While discrimination was occasionally mentioned, most barriers were more subtle, linked to the complexity of the system and the feeling that prevention was not a pressing priority or not yet available to them:

In Germany, for example, I don’t know what options there are. It seems very complicated, very complex. Which tests I could get, what I have access to. The information on such topics is mostly in German and in very technical language, so unfortunately, I don’t know much about it. That’s why in conversations with doctors, I feel inadequate or like I’m making a mistake. (LP, Türkiye, M34)

#### Type 3: Withdrawn or resigned (irregular participation)

Withdrawn participants were middle-aged, both men and women, often employed but not always happy with their working conditions. Their living circumstances were diverse, with partners or children, others alone. At the time of the interview, they had good or sufficient German language skills but acknowledged that low German language skills in the past had affected their healthcare experiences.

Many had faced health burdens, including cancer diagnoses, either since or after the migration to Germany. Yet these experiences did not draw them into prevention but rather reinforced withdrawal, as their encounters with the German healthcare system were often described in negative terms: they felt dismissed, not taken seriously, or discriminated against:

After all the experiences I’ve had, no, I don’t trust them. They [physicians] don’t take things seriously at all. A month ago, I was sick, but out of fear that the doctor would just say, ‘Drink tea,’ I didn’t go. It has already happened to me two or three times that I was seriously ill and the doctors didn’t help me. They just sent me home, and I came back in a worse condition. (LP, Iran, W45)

These experiences affected their trust in and perception of German physicians’ competencies. Despite knowing they could participate in regular CS even more frequently due to their health history, they deliberately did not at all or irregularly. Some leaned on health practices from their country of origin (e.g., consulting physicians via digital channels), self-medicated with shipped-in drugs or used home remedies. This was also echoed by several HCPs, who observed similar patterns in patient expectations and perceived quality of care:

In Syria, there are basically two systems. Public hospitals, where everyone goes for free treatment, but the care is often poor and uneven. And private clinics, where you pay everything yourself and in these clinics, you get all the tests and results in one day. So patients come from very different backgrounds. Someone who only knew the public system in Syria will be grateful just to receive care here in Germany. But someone who could afford private clinics there will often feel disappointed. Many of them think: ‘I work, I pay so much every month for insurance and this is all I get?’ It creates a feeling of being left behind, of not really being seen in this system. (HCP Urology, Syria, M)

#### Type 4: Marginalised (no participation)

Those assigned to the types of marginalised were exclusively women of different ages, all with several children and heavy caregiving responsibilities. They were usually not employed in paid work, financially dependent, and had very limited or basic German skills. Most lived in family households where traditional gender roles placed the burden of household and childcare squarely on their shoulders.

For them, CS was not part of their habits. They had little or no knowledge of preventive services, and preventive health had rarely been part of their upbringing:

The gynaecologist had a letter saying that I should do this test, this cancer test for women, but I didn’t go. I think I know if I’m healthy or sick. (LP, Afghanistan W35)

Their direct encounters with the German healthcare system were minimal, sometimes limited to a single consultation, often described as neutral or positive. Yet these contacts did not translate into participation in screening, because access was blocked by a lack of language skills (and translators), time, caregiving duties and some restrictions by male family members, especially in relation to gynaecological examinations, as both LPs and HCPs described:

Some families, for religious reasons, refuse to send their wives to a male doctor. (HCP General Practice, Iraq, W)

What I notice most here are the women who came as brides and don’t speak German. Some of them have been living here for years and still can’t speak the language. They are always dependent on their children or their husbands. That’s why they go to the doctor as little as possible and prefer Turkish doctors. Otherwise, they always need someone to go with them to translate. And as you know, translation is often not completely objective. Sometimes everything is said, sometimes a bit more, sometimes less, and often the husband adds his own opinion. (HCP General Practice, Türkiye, W)

Participants assigned to this group relied heavily on informal networks for information and language mediation, but these networks were rarely sufficient to enable access to preventive care. They generally did not mistrust the system but rather remained outside of it. Their non-participation was shaped by structural barriers and the heavy burden of daily chores and an under-prioritisation of their own (health) needs.

### Comparison and patterns across types

Across all four types, participation patterns are influenced by intersecting individual, social, and structural factors rather than single factors. Language proficiency and familiarity with the healthcare system appeared as particularly central elements. Participants who could communicate independently and who interacted more frequently with healthcare institutions through work, childcare, or personal illness described fewer barriers. In contrast, those with limited German skills and little system experience remained at the margins of preventive care, even when they expressed trust in medicine or interest in health.

Health burdens also shaped participation in distinct ways. Personal or familial illness experiences sometimes strengthened preventive orientation, but where such experiences coincided with low trust or perceptions of discrimination, they reinforced withdrawal. This pattern was most visible among those who had felt dismissed in previous encounters and who compared German care structures with systems in their countries of origin.

Gender differences were most pronounced among women with limited language skills, heavy care responsibilities, and limited autonomy (Type 4). Here, preventive care was virtually absent, reflecting how gendered caregiving roles intersected with linguistic and structural exclusion. In other groups, gender played a less defining role, suggesting that it acts through its intersections with other factors – particularly employment, and thereby social participation and German language acquisition, and household structure – and, in turn, supported engagement with health information and access to CS. Where such exposure was lacking, informal networks became the main source of health information and were influential for access to care.

## Discussion

This study used a qualitative, triangulated design to explore cancer screening (CS) participation among first-generation migrants from the Middle Eastern region in Germany. Combining interviews with laypersons (LPs) and healthcare professionals (HCPs) allowed us to examine participation from both experiential and professional viewpoints. The involvement of community researchers for interviews with LPs enabled trustful access to participants who might otherwise remain invisible to academic research. The analysis identified interdependent individual, social, and structural factors that shape (non-)participation (RQ1) and developed a typology of interacting factors associated with participation patterns (RQ2).

Our findings illustrate how participation in CS among first-generation migrants emerges at the intersections of individual, socio-cultural, economic, and institutional factors. These findings resonate with existing literature [4, 5], but also extend this insight by showing that many factors can act as both enablers and barriers. Religious values, for example, could facilitate preventive health orientation when interpreted as a God-given responsibility to care for one’s health [6, 28], yet al.so discouraged screening when fatalistic conceptions of illness prevailed [7]. Social relations likewise cut both ways [1]: informal translation and support from one’s networks supported participation, while collective decision-making, gendered norms, especially around gynaecological care, and caregiving responsibilities limited time and autonomy. Similarly, interactions with healthcare professionals were another decisive factor: positive encounters with HCPs and trust in their expertise could broaden exposure to CS participation, but also deepen institutional distrust and produce long-term disengagement when past discriminatory encounters eroded trust in them. The ambivalent and mutually entangled effects of these factors underscore their dynamic, relational embedding in structural, social, and symbolic power relations. It also points to the ways in which systemic racism and experiences of Othering permeate these levels, shaping healthcare experiences and institutional trust [29, 30].

Moreover, the study demonstrates that the interactions between systems and individuals are continuously negotiated through people’s reflections about healthcare encounters, e.g., as several participants explained their experiences at the time of the interview and after years of living in Germany were different than those during their first years [31]. Hence, this processual understanding of engaging in preventive behaviours and healthcare system engagements highlights the need to view CS not as a one-time decision but as shifting with individual priorities in life and broader acculturation processes.

In addition, our findings indicate that three interdependent conditions appear to be key to CS participation: (1) accessible communication and information about CS (i.e., understandable information, language skills or available language support), (2) procedural knowledge (knowing how referrals, entitlements and appointments work in the German healthcare system), and (3) institutional trust (positive encounters with healthcare providers, emotional safety and the experience of being taken seriously). In this respect, the results substantiate calls for greater attention to institutional/structural health system (communication) and organizational health literacy that account for prior system experiences and for both affective and procedural aspects of engagement [32, 33].

Typological approaches in qualitative health research [22, 23] offer a way to synthesise complex, intersecting influences. The typology developed here organises participation along a continuum from prevention-oriented to marginalized. Our four types – prevention-oriented, situational, withdrawn/resigned, and marginalized – map degrees of engagement and avoidance and capture participation as a situated practice, emerging from the interplay of structural conditions, social relations, and personal characteristics and experiences. While RQ1 captured the multi-level conditions that shape (non-)participation, the typology views them in relation to sociodemographic characteristics and hence social positions of the participants. It operationalises what intersectional approaches have long emphasised: that inequalities in health are not additive and emerge through the interaction of social location, institutional power, and lived experience [34, 35] as well as which social positions allow for mobilizing social and symbolic resources [26, 36].

These findings reaffirm the importance of capturing social heterogeneity beyond the broad category of “migration experience”, as discussed in the introduction. Without systematically including further variables (e.g., gender, German language proficiency, living situation, length of stay, etc.), it remains impossible to draw meaningful conclusions about the relationship between migration and health. More differentiated data are essential to identify how structural inequalities manifest across intersecting social positions and to inform targeted health research and policy [37].

Methodologically, the triangulated design enabled us to examine participation from two perspectives, i.e., laypeople and healthcare professionals, all with migration experience. The inclusion of community researchers was particularly valuable in building trust and facilitating access to laypeople participants who might otherwise remain excluded from academic research due to linguistic, social, or institutional barriers [31, 38]. Their linguistic and cultural proximity made it possible to reach individuals with limited German language skills or low institutional trust, as reflected in the fact that several participants chose to provide consent only under a pseudonym.

At the same time, recruitment by the community researchers inevitably shaped the data as they recruited people from their social and linguistic networks. The involvement of four individuals from different cities and community contexts helped ensure heterogeneity and contextual diversity. While not claiming to be representative, this reflects a deliberate methodological strategy to capture varied experiences within migrant populations through trust-based, situated access. In this way, the study exemplifies the value of participatory qualitative research for generating empirically grounded insights into preventive health behaviour among underrepresented groups [31].

Practically, the findings highlight the need for structural and communicative interventions that address both systemic and experiential dimensions of participation. First, accessible and culture-sensitive communication remains essential, for instance by acknowledging differences in prior healthcare system experiences [5, 9]. This may include materials for self-directed information use, such as leaflets or short videos, that allow individuals to engage with information at their own pace and level of familiarity. Furthermore, interactive formats that foster relational understanding are also required. Language mediators can play a pivotal role in this regard. Beyond linguistic translation, they act as cultural and relational intermediaries who contextualize information, build trust, and help patients navigate institutional procedures [26, 39].

Second, the findings highlight the need to strengthen the structural and organizational competence of healthcare providers and institutions. Efforts should aim to enhance how healthcare systems communicate, guide, and support diverse user groups [40]. This aligns with the concept of structural and organizational health literacy outlined in the National Action Plan Health Literacy, which emphasises institutional responsibility for creating transparent, responsive, and navigable systems [33].

### Limitations

Several limitations must be acknowledged. First, although the involvement of community researchers enhanced contextual understanding, their social positioning and networks shaped recruitment dynamics and the atmosphere of the interviews. The resulting sample thus reflects specific social and linguistic milieus among first-generation migrants from the Middle Eastern region in Germany. Second, the LP interview data were collected through multilingual interviews and partial translation, which inevitably involves subtle shifts in meaning and tone despite careful checking and collaborative interpretation. The study also relied on retrospective self-reports of experiences in the interviews that are embedded in personal memory and may be influenced by current life circumstances. Third, the interviews with HCPS offer valuable insights into professional experiences; however, they represent a selective group – physicians with their own migration experience and a declared interest in research participation. Perspectives of healthcare professionals without migration backgrounds are not included and this focus may have produced a more reflective and empathetic view of the described dynamics, while other perspectives within the healthcare system remain less visible. Last, the four types developed in the analysis constitute heuristic syntheses that help to organise and interpret recurring orientations toward cancer screening. They should not be understood as fixed or exhaustive categories. Boundaries between types are fluid, and some participants may share characteristics of more than one orientation. The typology thus serves as an analytical orientation that captures patterns across cases while remaining open to empirical variation and further refinement in future research.

## Conclusion

This study provides empirical insights into how participation in CS among first-generation migrants emerges from the dynamic interplay of individual, social, and structural factors. A typology developed here captures how social positions, shaped by gender, migration trajectories, and institutional encounters, shape the capacities and willingness to engage with CS. By integrating intersectional and socio-ecological perspectives, the study demonstrates how preventive behaviours such as CS are dynamic and changing and embedded in processes of prior experiences, trust, communication, and social position. Methodologically, the study highlights the potential of participatory, multilingual research to access underrepresented perspectives and make visible voices that often remain unheard in conventional health research. Practically, it calls for a paradigm shift toward institutional responsibility: healthcare providers and systems must develop structural and communicative competence that reflects the realities of diverse populations. Strengthening organizational health literacy and addressing systemic barriers are therefore central to fostering equitable, trust-based participation in preventive health services.

## Supplementary Information

Below is the link to the electronic supplementary material.

Supplementary Material 1

## Data Availability

The datasets used during the current study are available from the corresponding author on reasonable request.

## Abbreviations

CS: cancer screening
HCP: healthcare professional
LP: layperson
RQ: research question
