# Supplementary material for: Understanding cancer screening participation patterns among first-generation migrants in Germany: a typology based on the intersections of hindering and facilitating factors
Source: Int J Equity Health. 2026 Apr 4;25:96. doi: 10.1186/s12939-026-02843-w (PMC13067391; doi:10.1186/s12939-026-02843-w)
Supplement: Supplementary file 1 — Supplementary Material 1 [file 12939_2026_2843_MOESM1_ESM.pdf]

Interview guides

1) Laypeople

Introduction / Background

- Could you please introduce yourself briefly? How old are you, which country do you come from, and how long have you been in Germany?
- How do you live here in Germany – in shared housing, a refugee center, or in your own apartment/house?
- Do you have a partner or children?
- Did you learn a profession in your home country? Are you currently working in Germany or are you mainly taking care of the household and family?

Topic 1: Health behaviours

- Do you have specific rituals or daily habits to stay healthy?
    - What do you do for the health of your body?
    - What do you do for your mental health?
    - If you do not do anything specific for your health: what are the main obstacles that prevent you from doing so?
- Is there something you would like to do more for your health, but currently cannot?
- What is most important for you personally when you think about your health?
- Since you have been in Germany, have you had any health problems (physical or psychological) that you did not have before?
- If yes: what do you think are the reasons for these problems? Why do you think they did not occur earlier?
- When you have health problems: what do you do to make them better?
- Can you tell me a bit more about your experiences?
    - For example: Did you go to a doctor in Germany?
    - Did you search on the internet (German websites or in your native language)?
- What helped you? What is generally not helpful?
-

Preventive care and cancer screening

- Have you ever been to a doctor in Germany even though you were not sick? Can you tell me a bit more about that?
- Do you take part in regular health check-ups or preventive examinations?
- Have you participated in cancer screening (e.g. skin or cervical cancer)?
    - If yes: Which ones?
- The first time you went: did you know what would happen? Did you have enough information? What information was missing?
    - How often do you go to these examinations?
    - How do you feel during these examinations?
- If you have not taken part in cancer screening: can you tell me why not?
- If you would like to take part but have not done so yet: what are the reasons or barriers?
- What kind of help or support would make participation easier for you?
- What information do you need about these examinations?

Topic 2: Healthcare system in Germany

- Can you tell me how it is for you when you go to a doctor in Germany?

    - What do you appreciate in the German healthcare system? What not?
- Which barriers or obstacles do you face when you seek treatment?
- Are there things you feel you cannot tell a German doctor because they do not know or understand your culture?
- Do you usually go alone to the doctor, or do you take someone with you?
    - If yes: whom and why?

Trust and communication

- Do you generally trust doctors in Germany?
    - If yes, why?
    - If no: why not?
- What would you wish to be different in your interactions with doctors?
- Have you ever not taken a prescribed medicine or not followed medical advice? Why?
- How do you usually feel when you are at the doctor's office?

Information and support needs

- For people who are new to Germany: what information about the healthcare system should they receive?
- What kind of support would be important for them?

Topic 3: Life in Germany

- How has living in Germany influenced your physical and mental health?
- How has migration affected your ability to look after your health compared to your home country (for example, access to doctors, time for preventive care)?
- How has migration to Germany affected your relationships (partner, children, family back home)?
- Do you feel that women and men have different opportunities to take care of their health in Germany? In what ways?
- For you personally: is it easier or more difficult as a woman/man in Germany to go to the doctor or to take part in preventive care such as cancer screening? Why?
- How do family responsibilities (for example childcare, caring for relatives, household) affect your ability to use preventive health services?
- Who in your social environment (family, friends, community, online groups) supports you in questions of health or preventive care such as cancer screening?
- Who makes it more difficult, or discourages you from going?
- Do you have anything you would like to add we haven't talked about yet?

Healthcare professionals

Introduction

- Could you briefly introduce yourself: Which country are you originally connected to, how long have you lived and worked in Germany?
- What is your medical specialty and in which setting do you currently work (hospital, private practice, both)?
- Where did you complete your medical training and how many years have you worked in the German healthcare system?
- Which languages do you speak and in which do you offer medical consultations?

Topic 1: Patients and cancer screening

- To what extent do you encounter cancer and cancer screening (e.g. cervical, breast, skin, colorectal, prostate) in your professional practice?
- In your experience, what role does prevention or cancer screening play for patients with a first-generation migration background, specifically from the Middle Eastern region?
- Do you observe differences in knowledge, trust, or participation in cancer screening between patients with and without this migration background? Can you give examples?
- In your experience, how do patients with a migration background typically become aware of preventive services like cancer screening?
- Which factors do you think support their participation? Which factors hinder it?

Topic 2: Communication, trust, and barriers

- How do you experience consultations with patients with this migration background in general?
    - What challenges do you encounter (e.g. language differences, different understandings of health, expectations of the doctor–patient relationship)?
    - How do you deal with these challenges?
- What helps you to build trust with these patients?
- Do you sometimes use interpreters (professional or informal)?
    - If yes: how does this affect the conversation and mutual understanding?
    - Have you also used digital translation tools? With what results?
- Are there situations where communication barriers directly affect participation in preventive services or cancer screening?

Topic 3: Health information and access

- In your view, what makes health information on prevention and cancer screening “good” and helpful for patients with a migration background?
- Which channels or formats do you find effective (oral, written, digital, community-based)?
- Who could act as mediators or bridges between the healthcare system and migrant patients?
- Conversely, what do you consider less effective or even counterproductive?
- Can you describe a type of patient who, in your experience, has good access to preventive health information and screening?
- Can you also describe a type of patient for whom access is particularly difficult?
    - What role do factors such as social environment, networks, and structural/systemic factors play?

Topic 4: Professional perspective and personal experience

- In your view, do healthcare providers with a migration background have a particular role in prevention and cancer screening compared to colleagues without migration background?
- What should colleagues without a migration background know about working with migrant patients in this area?
- What should institutions and healthcare structures pay attention to in order to improve access to preventive services?
- From your own migration background and personal experiences: have you faced challenges in accessing health information in Germany (for yourself or family)?
    - If yes: how did you deal with them?
    - What would have helped in that situation?

Closing

- From your perspective: what would be the most important step to improve participation of people with a migration background in cancer screening in Germany?
- Is there anything else you would like to add that we haven't talked about yet?
